# Carrier Frequencies of Medically Actionable Pathogenic Variants in the Russian Population

**DOI:** 10.3390/ijms27125344

**Published:** 2026-06-13

**Authors:** Yulia Suvorova, Aleksandra Monakhova, Nikolay Chekanov, Olga Musharova, Elizaveta Moskovkina, Igor Zaigrin, Ivan Antonov, Olesia Klimchuk, Dmitry Pustoshilov, Daria Zorina, Evgeny Klimuk, Konstantin Severinov

**Affiliations:** Biotech Campus Limited Liability Company, 117437 Moscow, Russia

**Keywords:** whole-genome sequencing, WGS, secondary findings (SF), carrier frequency, ACMG

## Abstract

Genomic sequencing can reveal potentially life-threatening clinically actionable secondary findings in healthy individuals. Little is known about the spectrum and frequency of secondary findings in healthy people in Russia. Here, we analyzed whole-genome sequences of 42,826 healthy volunteers from urban populations across Russia, focusing on known pathogenic and likely pathogenic variants of 81 genes associated with treatable or preventable monogenic diseases listed in the American College of Medical Genetics and Genomics’ Secondary Findings recommendations (ACMG SF v3.2). Based on the ClinVar 20250421 version, secondary findings were detected in 1186 (2.76%) participants. Cancer phenotypes were the most common category of secondary findings present in 565 (1.32%) participants, followed by cardiovascular phenotypes (454 individuals, 1.05%). Genes harboring the most frequent variants were *BRCA1* (151 variants), *BRCA2* (100), *RYR1* (93), and *LDLR* (71). In addition, we found 238 potential loss-of-function variants in dominant ACMG SF v3.2 list genes in 280 (0.65%) participants, which, if confirmed by orthogonal methods, could increase the frequency of secondary findings to 3.41%. A study of such depth and scale was performed for the first time in the Russian population.

## 1. Introduction

Several large-scale national genomic projects, including, for example, the UK Biobank [1], AllOfUs [2], FinnGen [3], the Qatar Genome Project [4], and Korea4K [5], are being carried out throughout the world. Typically, they aim to aggregate tens of thousands of whole-genome sequences of people from a particular country, supplemented, whenever possible, with personal health and clinical data. By filling the gaps in known human genetic variation and allowing to determine the frequencies of specific genetic variants, such projects facilitate clinical interpretation of genomic data worldwide.

In Russia, a “National Genetic Initiative” (NGI) population genomic program was launched in 2023 and reached its target of 100,000 whole-genome sequences in late 2025. About 40% of volunteers participating in NGI were healthy individuals from the general urban adult population in Moscow, St. Petersburg, Ryazan, Krasnodar region, Samara region, Tyumen region, Krasnoyarsk region, Bashkortostan, Khanty-Mansiysk, and the Udmurt Republic.

In clinical practice, genome sequencing data analysis can reveal genetic variants that predispose a patient to health conditions different from the initial indications. The same can happen in the context of a scientific study that concentrates on participants who are considered to be generally healthy. The American College for Medical Genetics and Genomics (ACMG) has published recommendations for clinical sequencing laboratories to report such incidental secondary findings (SF) to patients if the variants reside in genes with substantial risks of monogenic diseases that can be treated or prevented. The ACMG v3.2 version of the reportable secondary findings list [6] includes 81 genes. According to ACMG recommendations, all pathogenic and likely pathogenic variants in most of these genes should be reported. The only exceptions are the *TTN* and the *HFE* genes, for which, respectively, only truncating variants and a single rs1800562 variant are reportable.

Previous studies of ACMG-listed genes in Russia were limited either by the size of the cohort studied [7,8,9] or the genes included [10]. To fill this gap, we here present the result of a comprehensive secondary-findings screening of 42,826 whole genomes collected across Russia.

## 2. Results

### 2.1. The Cohort and Approach

The cohort of this study was a group of 42,826 unrelated, generally healthy adult volunteers (no clinical examination was performed) residing in several major urban areas across Russia, aged 18–89 years (median age = 39); 61% of participants were female (see Supplementary Figure S1). All participants completed a questionnaire on personal and family health, lifestyle, and self-identified ethnicity. In total, 60.5% considered themselves Russians. Other most frequent self-reported ethnicities were Tatars (8.6%), Bashkirs (2%), Ukrainians (1.4%), Udmurts (1.3%), Chuvash (0.92%), Armenians (0.54%), and Mari (0.45%). Among mixed self-reported identities, the most frequent were Russians/Ukrainians (0.53%) and Bashkirs/Russians (0.53%). We could not assign self-reported ethnic backgrounds for about 15% of volunteers due to the ambiguity of their answers. Supplementary Figure S2 shows a PCA plot comparing the NGI participants with the 1000 Genomes samples, demonstrating substantial overlap with non-Finnish Europeans, Finns, Americans, and East Asians, which is consistent with Russia’s geography and ethnic diversity.

Our pipeline for genetic variant identification is presented in Supplementary Figure S3. We concentrated on known pathogenic (P) and likely pathogenic (LP) ClinVar-annotated variants in 81 genes from the ACMG secondary findings list v.3.2. We considered an individual as a “Genotype-Positive Participant” (GPP) if either a P/LP heterozygous variant associated with a dominant disorder or a homozygous variant/two variants in a compound heterozygous state in recessive genes were identified in their genome. When a recessive gene variant in a heterozygous state was observed, the participant was considered a carrier (CP) (see Section 4 and Supplementary Figure S1 for details). All P/LP variants found in GPPs were manually checked and inspected using Integrated Genome Viewer (IGV). By applying strict criteria described in Section 4, we also identified new (not listed in ClinVar) potentially loss-of-function (pLOF) variants in dominant genes.

### 2.2. Overall Statistics of Secondary Findings

**Figure 1:**
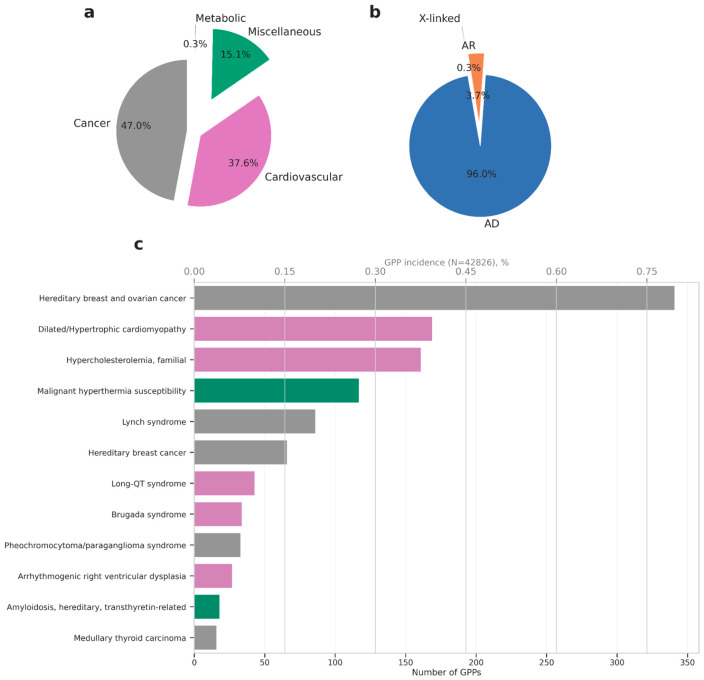
Overall statistics of secondary findings among the National Genetic Initiative participants. Distribution of GPPs over types of inheritance (**a**) and ACMG SF v3.2 phenotype categories (**b**). Incidence of GPPs with the most frequent phenotypes (**c**). ACMG SF phenotypes are color-coded as in panel (**a**).

Secondary findings were identified in 1186 (2.76%) individuals from our cohort. According to ACMG SF phenotype classification, 565 (1.32%) GPPs had known P/LP variants in genes associated with cancer phenotypes; 454 (1.05%)—with cardiovascular phenotypes; four—in genes associated with inborn errors of metabolism; and 181 (0.42%)—in genes associated with Miscellaneous phenotypes (Figure 1a). A total of 21 participants had more than one reportable variant, the most common combination being the simultaneous presence of cancer and cardiovascular SFs. The frequency of secondary findings with cardiovascular phenotypes matches the recent findings from an independent Russian cohort [10].

A total of 1142 GPPs had 492 distinct P/LP variants in genes related to autosomal dominant phenotypes, 46 had two P/LP variants (12 distinct variants) in the same gene associated with autosomal recessive phenotypes, and three GPPs had two distinct variants in genes associated with X-linked phenotypes (Figure 1b). Additional testing is required to resolve the phase in cases where two different P/LP variants in the same gene were found. The age and gender distribution of carriers corresponds to that of the entire cohort (Supplementary Figure S4). The most frequent phenotypes were breast and ovarian cancer, hypertrophic and dilated cardiomyopathy, familial hypercholesterolemia, predisposition to malignant hyperthermia, and the Lynch syndrome (Figure 1c).

We did not encounter any ClinVar P/LP variants in the following ACMG SF v3.2 list genes: *ACTA2*, *ACTC1*, *ACVRL1*, *CALM1*, *CALM2*, *CALM3*, *DSC2*, *ENG*, *MYL2*, *MYL3*, *NF2*, *PRKAG2*, *PTEN*, *RBM20*, *SDHAF2*, *SMAD3*, *SMAD4*, *STK11*, *TGFBR1*, *TMEM43*, *TMEM127*, *TNNT2*, *TPM1*, *TSC1*, *WT1*. For most of these genes, the most common known pathogenic variants have allele frequencies in the non-Finnish European (NFE) gnomAD cohort of 0.00001 or less, which may explain the lack of carriers in our cohort. However, the *PTEN*:c.802-2A>T (rs587782455) variant has a frequency of 0.0001 in NFE and thus could have been observed. Upon closer examination of the genomic context, we noticed that *PTEN*:c.802-2A>T (rs587782455) is immediately adjacent to a low-complexity region with a 15 bp polyT tract. The gnomAD marks this variant as “failing allele-specific VSQR filter,” and most carriers have an allele balance of 0.2–0.3. In fact, in one volunteer from our cohort, we detected *PTEN*:c.802-2A>T (rs587782455) supported by three reads (on both strands), while the reference allele was supported by 22 reads (allele balance of 0.12 leading to a homozygous reference call). Thus, *PTEN*:c.802-2A>T (rs587782455) may have limited discoverability due to technical reasons.

### 2.3. Frequencies of P/LP Variants in ACMG v3.2. Genes Related to Autosomal Dominant Phenotypes

**Figure 2:**
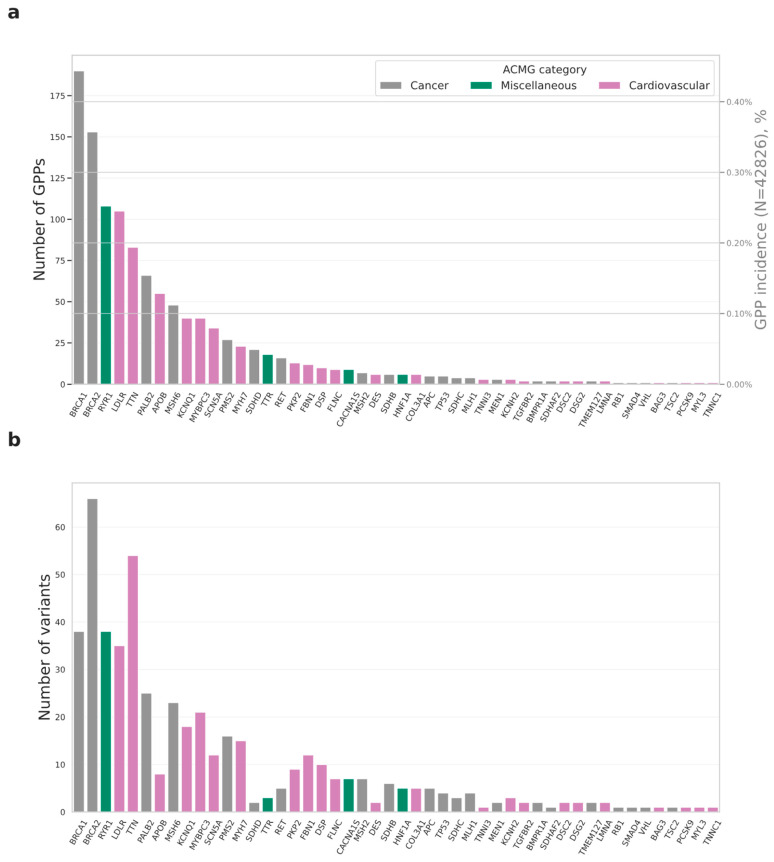
Statistics of SF in genes associated with autosomal dominant conditions. Numbers of GPPs with P/LP variants in ACMG SF v3.2 genes associated with preventable dominant conditions (**a**) and the numbers of variants in ACMG SF v3.2 genes (**b**) are presented.

Among 1142 GPPs with SFs in autosomal dominant genes, the most frequent genes were *BRCA1* (190 GPPs, 38 P/LP distinct variants) and *BRCA2* (153 GPPs, 66 variants) genes associated with hereditary breast and ovarian cancer; the *RYR1* gene associated with susceptibility to malignant hyperthermia (108 GPPs, 38 variants), the *LDLR* gene associated with familial hypercholesterolemia (105 GPPs, 35 variants) (Figure 2a,b). Predictably, among the genes associated with dilated (DCM) or hypertrophic (HCM) cardiomyopathies, *TTN*—the longest human gene—had the most variants. When considering only LOF variants as per ACMG recommendations, 54 distinct *TTN* variants in 83 GPPs in our cohort were detected.

### 2.4. Frequent Variants of Autosomal Dominant Genes

**Figure 3:**
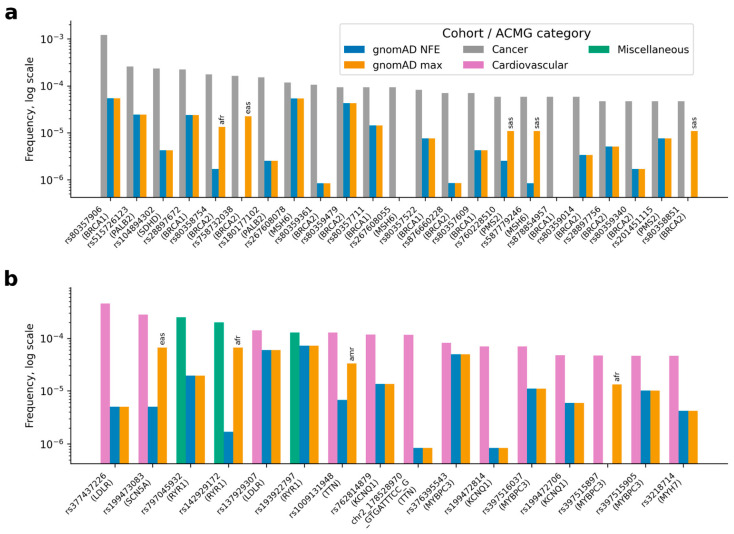
ClinVar-pathogenic variants in dominant ACMG SF v3.2 genes found in ≥4 participants and with frequencies higher than in any gnomAD cohort (log-scaled). (**a**) Frequent variants with cancer-related phenotypes. (**b**) Frequent variants with non-cancer phenotypes. The “gnomAD_max” label represents a cohort with the maximal frequency of a given variant (eas—East Asian, afr—African/African American, amr—Admixed American, sas—South Asian). Variants are named by their dbSNP IDs (when available) or in the “Chromosome_Position_ReferenceAllele_AlternativeAllele” format.

Among the 492 distinct variants in genes associated with dominant diseases, 356 (72%) were encountered just once (singletons), 85 (17%)—2–3 times, and 51 (10%) ≥ 4 times. Of the 52 P/LP variants that occurred ≥ 4 times in our cohort (Supplementary Table S1), 39 have frequencies that are higher than in any gnomAD cohort (Figure 3a,b). Most of such “frequent” variants specific to our cohort were predominantly or exclusively encountered in people self-identified as Russians (which constitute most of our volunteers) and may indicate frequencies specific to the Russian and/or Slavic populations. Among the frequent cancer-related variants, 15 were in the *BRCA2* and *BRCA1* genes (9 and 6, respectively). The most frequent BRCA variants include well-known Slavic founder *BRCA1* mutations rs80357906 (c.5266_5267insC) and rs80357711 (c.4035del), and the *BRCA2* mutation rs80358754 (c.5286T>G) [11]. Two variants in the *PALB2* gene, rs180177102 (c.1592del) and rs515726123 (c.509_510del), associated with breast cancer, were previously found in breast cancer patients from Russia [12]. Another frequent variant in our cohort, rs104894302 (c.305A>G) in the *SDHD* gene associated with hereditary paraganglioma-pheochromocytoma syndrome, was previously described in three patients of Slavic ancestry [13] in Russia. Among 20 GPPs with the rs104894302 variant, 17 identified themselves as Russians (others were of mixed ethnicities).

Some variants were prevalent in self-identified Tatars, Bashkirs, or other ethnicities from the Volga region. Among the 39 GPPs with the most frequent variant rs377437226 (c.2546C>A) in the *LDLR* gene associated with familial hypercholesterolemia, 20 identified themselves as Tatars or Bashkirs; this variant was not previously reported in Russia. The rs28897672 (c.181T>G) variant in *BRCA1* (found in 19 GPPs) and the rs758732038 (-39-1_-39del) variant located in the 5′UTR of *BRCA2* (found in 14 GPPs) were the most frequent cancer-related variants among participants who identified themselves as Tatars and Bashkirs. These variants were previously detected in Tatar and Bashkir women diagnosed with breast or ovarian cancer [14]. The rs199473083 (c.845G>A) variant in the *SCN5A* gene associated with the Brugada syndrome 1 was found in 17 GPPs, all of whom identified themselves as Tatars or Bashkirs. This variant was not previously reported in Russia. Other frequent variants previously unreported in Russia included rs267608055 (c.1299T>G) associated with the Lynch syndrome and rs111888148 (c.1589G>A) associated with the malignant hyperthermia found, correspondingly, in 8 and 11 GPPs who self-identified as Tatars or Bashkirs. The *BRCA2* variant rs80359361 (c.2899_2900del), also previously unreported in Russia, was found in nine GPPs, of which six identified themselves as Udmurts.

To determine if SFs in autosomal dominant genes have an impact on participants’ health, we analyzed questionnaires of 42,596 participants, excluding those incompletely filled, as well as those of carriers of recessive SFs and pLOF variants in cancer-associated genes. Questionnaire data were available for 560 GPPs with SFs in genes associated with autosomal-dominant oncological phenotypes (two carriers were excluded due to incomplete questionnaires). Across the whole 42,596 participant cohort, 1011 participants reported a history of cancer. A total of 966 out of 42,036 participants without oncological SFs reported cancer history (2.30%). Among carriers of oncological SFs, cancer was reported by 45 of 560 individuals (8.04%). The difference between the two groups was highly significant by the χ^2^ test (χ^2^ = 76.0603, *p* = 3.01 × 10^−18^). In a logistic regression model adjusted for age and sex, the presence of a cancer-category SF was associated with increased odds of reporting cancer: OR = 3.8794, 95% CI 2.8219–5.3333, *p* = 6.92095 × 10^−17^. Thus, carriers of these SFs had roughly 3.9-fold higher odds of having a cancer diagnosis compared with participants without such findings. A detailed clinical assessment would help to refine these numbers.

### 2.5. Frequencies of P/LP Variants in ACMG Genes Related to Autosomal Recessive and X-Linked Phenotypes

In our cohort, there were 46 GPPs with two variants in the same gene associated with an autosomal recessive phenotype (39 homozygotes and seven probable compounds). In addition, there were 4117 (9.6%) heterozygous CPs with only one of 190 unique P/LP variants in such genes (Supplementary Table S2), among them 295 carried variants in more than one gene. Three GPPs had P/LP variants in genes associated with X-linked phenotypes.

A total of 38 GPPs were homozygous for rs1800562 (c.845G>A), the only reportable variant in *HFE*, a gene associated with type 1 hemochromatosis; 2339 volunteers were heterozygous carriers of this variant. The observed allele frequency in our cohort (2.8%) is significantly lower than that in non-Finnish Europeans (NFE, 7.1%). Our results are in accordance with the previous report of Adler et al. [15], which estimated the rs1800562 frequency in the eastern Slavic populations at around 3%. The higher gnomAD NFE counts could be attributed to overrepresentation by Western and Northern European ethnicities.

One GPP was homozygous for the rs76151636 (c.3207C>A) variant in the *ATP7B* gene associated with Wilson disease. Another was a potential compound carrier of rs76151636 and reportable *ATP7B* variant rs587783299 (c.1877G>C). Among the 732 carriers of P/LP variants in *ATP7B*, 414 had the rs76151636 variant, yielding an allele frequency (AF) of 0.5%. This is significantly higher than the frequency in NFE (AF = 0.1%) and equal to the highest reported AF value in Ashkenazi Jews. A previously reported *ATP7B*:c.[3948del;3942_3943del] haplotype (rs1057516228 and rs1057516227 in *cis* phase) was observed in 18 CPs. 13 of them self-identified as Tatars or Bashkirs. Consistently, this haplotype was previously described in the Bashkir population [16].

Three GPPs carried two different compound variants in the *MUTYH* gene linked to autosomal recessive colorectal adenomatous polyposis. In each case, one variant was rs36053993 (c.1103G>A), while the second variant differed (rs140342925, rs587781864, or rs34612342). The individual with rs36053993 and rs587781864 has a grown-up child, who participated in the Initiative but was not included in the SF study because of being a first-degree relative. This individual carries only the rs36053993 variant, confirming the in-trans configuration in the parent. Overall, 469 individuals were carriers of P/LP variants in this gene, with the rs36053993 variant being the most frequent (AF = 0.3%), which is lower than in Europe (NFE AF = 0.6).

There were 139 carriers of other P/LP variants in *BTD*. In addition, 3138 CPs were heterozygous for the rs13078881 (c.1270G>C, p.Asp424His, sometimes reported as c.1330G>C p.Asp444His) *BTD* variant, and 122 had it in the homozygous state, which is known to be benign and is commonly encountered worldwide [17]. The rs13078881 variant causes biotinidase deficiency in a compound heterozygous state with any other pathogenic variant of *BTD*. We found two GPPs with possible compounds of the rs13078881 variant with a pathogenic haplotype c.[40_41del;c.44_45del] (rs765906887 and rs750965140 variants). These GPPs may thus be at risk of developing biotinidase deficiency. A grown-up child of one participant took part in the Initiative but was not included in the SF study because of being a first-degree relative. This individual carries only the c.[40_41del;c.44_45del] haplotype, confirming the in-trans configuration in the parent. We also identified 106 CPs with the c.[40_41del;c.44_45del] haplotype only. This haplotype is sometimes reported as a compound heterozygote [18], probably erroneously, given that the two variants are very close to each other.

One GPP had two different pathogenic variants in the *GAA* gene associated with Pompe disease (rs386834236, c.-32-13T>G and rs374470794, c.1802C>G), and 496 participants had P/LP variants in this gene. There were also 58 P/LP carriers of pathogenic variants in the *RPE65* gene (RPE65-related retinopathy), 29 carriers of P/LP variants in the *CASQ2* gene, and five in the *TRDN* genes (associated, correspondingly, with catecholaminergic polymorphic ventricular tachycardia 2 and 5) (Supplementary Figure S5).

Overall, we found seven GPPs with probable compound heterozygous variants (Supplementary Table S3). In two of these cases, the individuals have grown-up children who inherited only one of the two variants, thereby confirming the compound status. For the remaining cases, additional studies are required to establish the in-trans configuration.

Three GPPs had P/LP variants in genes associated with X-linked phenotypes. One female participant carried the rs72554308 (c.119G>A) variant in the *OTC* gene related to ornithine transcarbamylase deficiency. Two others (a male and a female) were carriers of rs28935197 (c.644A>G) in the *GLA* gene associated with Fabry disease. In the metabolic disorders group, late-onset Pompe and Fabry diseases deserve particular attention as timely genetic diagnoses enable disease-specific treatments. For Fabry disease, this includes enzyme-replacement therapy (ERT) and oral chaperone therapy with migalastat [19]; for Pompe disease, intravenous ERT with recombinant human GAA [20].

### 2.6. Carrier Frequencies of Potential Loss-of-Function Variants in Dominant ACMG SF Genes

**Figure 4:**
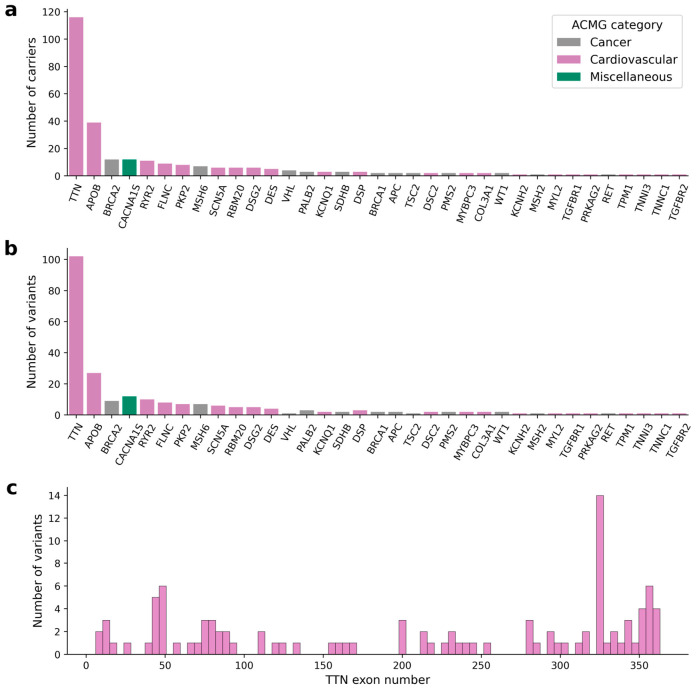
Potentially pathogenic variants in the ACMG SF v3.2 genes. Number of pLOF variant carriers (**a**) and pLOF variants (**b**) in ACMG v3.2 genes associated with autosomal dominant phenotypes. (**c**) Distribution of pLOF variant numbers across the *TTN* gene exons.

We next searched for potential loss-of-function (pLOF) variants in dominant ACMG v3.2 genes that (*i*) are not present in the ClinVar (20250421 version) and (*ii*) have an AF ≤ 0.01 in gnomAD (see Section 4). Because pLI (and similar constraint metrics) are based on the ratio of observed to expected variation in gnomAD, pathogenic alleles for late-onset disorders (as in our study) may still be observed in population databases, making these metrics not applicable for our analysis. Genes where a loss of function is not the mechanism of disease (*TTR*, *RYR1*, *PCSK9*, *MYH7*) were excluded from this analysis. In total, 238 variants in 280 participants were found (Figure 4a,b, and Supplementary Table S4). Most pLOF variants were in genes associated with cardiovascular diseases (193 unique variants were found in 226 participants (81%)), with the *TTN* gene having the largest number of variants and carriers (102 unique variants, 116 carriers). The distribution of pLOF variants across the *TTN* exons is presented in Figure 4c. The highest count of variants was observed in exons 326,358 (located in the A band region), and 48, which corresponds to previously reported *TTN* hotspots [21].

There were five “frequent” pLOF variants encountered in ≥4 of our volunteers (Table 1). All carriers of pLOF variants *APOB*:c.6256C>T and *TTN*:c.106571dup reported their ethnicity as Russian. Carriers of a pLOF frameshift variant in the *APOB* gene (c.4773del) identified themselves as Chuvash or Mari, while all carriers of a new stop-gained variant in the *BRCA2* gene (c.2521A>T) identified themselves as Udmurts.

We performed manual IGV review of recurrent pLOFs listed in Table 1 to confirm adequate allele balance, mapping quality, and strand representation. No alignment artifacts were observed. We next examined the questionnaires of participants who carried these pLOF variants. Among the four carriers of the stop-gain variant in the *VHL* gene (associated with von Hippel-Lindau syndrome), one reported cancer in one of his parents, as did one of the four *BRCA2* pLOF variant carriers.

All pLOF variants reported here should be considered candidate findings and require orthogonal confirmation. Analysis of questionnaire data for pLOF variant carriers in genes associated with oncological diseases is constrained by a small sample size. Only 31 such carriers were identified in the cohort, and just two reported a personal history of cancer. These low numbers provide insufficient statistical power, so comparative tests did not reach significance.

## 3. Discussion

Secondary findings’ frequencies derived from several large genomic projects are currently available. For example, a Singapore study by the SG10K_Health project evaluated 9051 whole genomes of diverse ancestries (61% Chinese, 21% Indians, 18% Malay) over 73 genes from an ACMG SF v.3.0 list [22] reportable SFs in 3.4% of participants. A recent study by the Qatar Genome Program covered 78 genes from the ACMG SF v.3.1 list in 14,392 whole genomes of Qataris and yielded a reportable SF frequency of 3.5% [4]. In the Indian cohort of 500 families, the SF frequency was 2.2% [23]. In similar studies by other genomic consortia using different sequencing strategies, different ACMG SF list versions (some included extra genes), and cohorts of varied size, ethnicity, and clinical status, proportions of individuals with secondary findings were reported between 2% and 5% (Table 2).

The goal of this work was to estimate the frequency of clinically relevant P/LP variants in a large Russian population cohort. We analyzed variants in 81 ACMG SF v3.2-listed genes in 42,826 unrelated participants scattered throughout several major Russian cities. 2.76% of participants carry at least one pathogenic or likely pathogenic variant associated with autosomal dominant diseases from the ACMG SF v3.2 list or two variants in genes associated with recessive disorders, while 9.6% participants are carriers of recessive variants. These frequencies broadly correspond to reported SF frequencies in other populations.

The reported variants and their frequencies reflect the specific versions of the ACMG SF list and ClinVar annotation used in the study and may change as these resources are updated. We have checked 100 most frequent SF variants for the presence of an up-to-date ClinGen [30] classification as of June 2026 (Supplementary Table S1). For 45 variants, no ClinGen classification was available, reflecting the currently limited scope and depth of this database. Among the 55 variants with available ClinGen classification, most were concordant with the ClinVar (20250421 version) classification or remained within the same Pathogenic/Likely pathogenic spectrum. The only exception was rs376395543 in *MYBPC3*, which was classified as Pathogenic/Likely pathogenic in ClinVar but as Uncertain significance in ClinGen. The most complete ClinGen coverage was observed for the *BRCA1* and *BRCA2* genes. For these genes, ClinGen classification was absent only for 6 out of 34 variants (rs397509279, rs80357094, and chr17_43094221_TGAGGATC_T in BRCA1; and rs80358754, rs758732038, and rs80359405 in *BRCA2*). Overall, we conclude that ClinGen is an important but incomplete source of expert validation for ClinVar classifications. Significant expansion of ClinGen with curated variants will be necessary to reduce uncertainty and improve consistency in variant classification.

Malignant hyperthermia is a rare but potentially fatal pharmacogenetic muscle disorder triggered in susceptible patients by inhaled halogenated anesthetics or succinylcholine. Although exact statistics for Russia are not available, individual clinical cases have been reported [31]. 85 participants from our cohort carry pathogenic or likely pathogenic variants in the *RYR1* or *CACNA1S* genes. The frequency of the *RYR1* variants c.2505del and c.1589G>A significantly differs from frequencies reported for other populations (Figure 3b).

We have observed that frequencies of some variants in genes associated with breast and ovarian cancer appear to have a strong connection with carrier ethnicity. We found a set of variants related to different phenotypes that are specific to participants who identified themselves as Tatars and Bashkirs; some of them were previously detected among cancer patients [14], but most were not reported previously in Russia. The high frequency of the *BRCA2* rs80359361 among participants who identified themselves as Udmurts has previously not been reported. We also found several frequent potential ethno-specific variants associated with cardiovascular diseases. A limitation of our analysis is the reliance on self-reported ethnicity rather than genotype-inferred ancestry, since self-identification may not reflect underlying genomic background. Incorporating genetic-ancestry inference in future work would improve adjustment for population stratification and is essential for confirming ancestry-specific variants and uncovering potential founder effects.

Our analysis of pLOF variants revealed a much higher overall percentage of those related to cardiovascular phenotypes (81%) and a much lower percentage of cancer-related phenotypes (15%) than among the known P/LP variants (38 and 47%, respectively). While all potentially pathogenic variants will require confirmation via functional analysis and/or segregation analysis in families with signs of disease, the result may be explained by the fact that variants related to cardiovascular phenotypes are much understudied compared to cancer-related ones.

Informing a person about the discovered genetic risks of a pathological condition is an important step towards personalized medicine. There are different practices about the procedure for returning secondary findings in different countries [32,33]. Official recommendations on SF reporting in Russia concern only patients who have been referred to genetic testing for other medical reasons [34]. Further, according to Russian legislation, each person has a right to receive information about their health, but this information cannot be provided against the person’s will. All participants of NGI have an opportunity to realize this right and decide whether they prefer to receive information about detected SF by signing a corresponding section in their informed consent (90% of participants indicated such a preference). All GPPs who agreed to receive information about their SFs were contacted and asked to reconfirm their decision. For those who agreed, a written report summarizing the genetic findings was prepared and delivered to a clinical geneticist. Clinical geneticists who reviewed the reports conducted in-person or remote consultations, interpreting the findings in the context of participants’ family history, comorbidities, and current health status. Based on these assessments, individualized clinical recommendations were provided. Where appropriate, these included additional diagnostic testing, follow-up, and measures for prevention or early detection of associated conditions. As of the time of this writing, 43% of GPPs agreed to undergo a clinical geneticist consultation (32% have received it), and 20% were referred to healthcare institutions for further monitoring and treatment.

## 4. Materials and Methods

### 4.1. Cohort Description

All NGI participants signed an informed consent and gave a written permission for repeated contacts and for a reception of information about their newly discovered genetic risks. The participants had completed a questionnaire to report on their own and familial health conditions, lifestyle choices including diet and alcohol consumption, and self-described ethnicity, both their own and that of each of their grandparents.

### 4.2. Sequencing

Genomic DNA was extracted using the magnetic bead-based sorption method (MGIEasy Magnetic Beads Blood Genomic DNA Extraction Kit, MGI, Shenzhen, China) and subsequently used for the preparation of genomic libraries for sequencing. Library preparation was performed using a PCR-free protocol with enzymatic DNA fragmentation (MGIEasy FS PCR-Free Library Prep Set, 96 reactions (MIX), MGI). Prepared libraries were sequenced on the (DNBSEQ-T7 sequencer (PE150), Shenzhen, China) with a target median coverage of 30×. Only samples yielding 30× or higher median coverage were included in the study cohort.

### 4.3. Bioinformatics Analysis

Reads were passed to cutadapt 4.2 [29,35] for adapter removal (MGIEasy DNA Adapters, MGI) and trimming of low-quality read ends. Mapping to the GDC reference genome GRCh38.d1.vd1 was performed with bwa 0.7.17 [36]. Duplicate marking was performed with Picard 2.27.5. Short variation calling was performed with DeepVariant 1.4 [37] using the “Variant Reads” selector model, MAPQ ≥ 10 filter, and with tracking of reference-supporting reads enabled. Joint calling was performed with GLNexus 1.4.1 [38] using a “DeepVariantWGS” configuration preset to produce a single multisample VCF file for 81 ACMG SF v3.2 [6] genes. Indel normalization, splitting of multiallelic sites, and call filtering (GQ ≥ 20, DP ≥ 10) were performed with bcftools 1.21 [39]. Variant annotation was performed with the Ensembl Variant Predictor (VEP v115.1 [40]), including information from ClinVar 20250421 [41], gnomAD v.4.1 [42], SpliceAI v1.3.1 [43], and AlphaMissense v1.0 [44]. Structural variants were not analyzed. Kinship inference was performed with KING 2.2.7 [45] after the conversion to Plink format (Plink 1.90 [46]); all MZ- and 1st-degree relationships were excluded via the removal of the least possible set of sample-vertices participating in relationship-edges in a global relationship graph. PCA was performed on a joint dataset that additionally included the HGDP + 1KG dataset [47,48] (packaged by gnomAD v3.1.2 [42]). Merging, common-variant selection, and basic QC on the joint dataset were performed with bcftools 1.21 [37] using ≥90% sample and variant call rate thresholds.

### 4.4. Variant Classification and Interpretation

Variants annotated as Pathogenic or Likely Pathogenic and with a ≥1-star status and no conflicts in ClinVar and related to the described conditions were designated as known pathogenic variants (P/LP). As per ACMG SF recommendations, for the *TTN* gene, only truncating variants were included, and for the *HFE* gene, only one variant was included (rs1800562). We considered an individual a Genotype-Positive Participant (GPP) if a P/LP heterozygous variant was found in dominant genes, or a homozygous variant or two variants in a compound heterozygous state in recessive genes. If only one heterozygous variant was found for recessive genes, we considered the participant a carrier (CP).

To find potential loss-of-function (pLOF) variants at the first step, variants present in ClinVar were excluded. Then we kept only variants found in dominant genes in which loss of function is a known mechanism of the disease that had gnomAD AF ≤ 0.01. Variants with potential loss of function (frameshifts, stop-gains, start-losses, splicing alterations) that were predicted to escape nonsense-mediated decay (NMD, as per rules in [49]) were included. We considered an individual a Potentially Positive Participant (PPP) if such a variant was found in their genome.

## Figures and Tables

**Table 1:** The most frequent pLOF variants in dominant ACMG SF v3.2 genes.

Variant	Gene	Consequence	HGVSc	N GPPs	AF	gnomAD Max Frequency	Ethnicity (Self-Identified)
rs943553766	*APOB*	stop-gained	c.6256C>T	5	0.000059	0	Russian
rs1663340804	*APOB*	frameshift variant	c.4773del	5	0.000059	8.48 × 10^−7^	Chuvash/ Mari
chr2_178529179_C_CT	*TTN*	frameshift variant	c.106571dup	5	0.000059	0	Russian
chr3_10141941_G_T	*VHL*	stop-gained	c.94G>T	4	0.000048	0	Russian
chr13_32336876_A_T	*BRCA2*	stop-gained	c.2521A>T	4	0.000048	0	Udmurts

**Table 2:** Frequencies of secondary findings in different populations.

Project	SF Carriers, %	Method	Sample Size	ACMG Version	Number of Genes	Country, Ethnicity	Citation
UK Biobank	2	WES	49.960	v2.0	59	UK, Mixed	[24]
India	2.2	WES	500 families	v3.2	81	India, South Asian	[23]
SG10K Health project	2.63	WGS	9.051	v2.0	59	Singapore, Mixed Asian	[22]
Netherland cohort	2.7	WES	1640	v2.0	59	Netherland, European	[25]
Pakistan cohort	2.7	WGS	863	v3.1	78	Pakistan, South Asian	[26]
Qatar Genome Program	3.5	WGS	14.392	v3.1	78	Qatar, Arabian	[4]
DiscovEHR	3.5	WES	50.726	v1.0	76 (56 ACMG)	USA, Mixed	[27]
Greek cohort	4.2	WES	280	v3.2	81	Greece, European	[28]
CSVS	5.0	WGS/ WES	1129	v3.1	78	Spain, European	[18]
DISCO	5.3	WES	2987	v3.0	73	China, Asian	[29]

## Data Availability

The original contributions presented in this study are included in the article/Supplementary Material. Further inquiries can be directed to the corresponding authors.

## Supplementary Materials

The following supporting information can be downloaded at: https://www.mdpi.com/article/10.3390/ijms27125344/s1.

## References

[1] Bycroft C., Freeman C., Petkova D., Band G., Elliott L.T., Sharp K., Motyer A., Vukcevic D., Delaneau O., O’Connell J. (2018). The UK Biobank Resource with Deep Phenotyping and Genomic Data. Nature.

[2] Bick A.G., Metcalf G.A., Mayo K.R., Lichtenstein L., Rura S., Carroll R.J., Musick A., Linder J.E., The All of Us Research Program Genomics Investigators, Manuscript Writing Group (2024). Genomic Data in the All of Us Research Program. Nature.

[3] Kurki M.I., Karjalainen J., Palta P., Sipilä T.P., Kristiansson K., Donner K.M., Reeve M.P., Laivuori H., Aavikko M., Kaunisto M.A. (2023). FinnGen Provides Genetic Insights from a Well-Phenotyped Isolated Population. Nature.

[4] Elfatih A., Saad C., Mifsud B., Mbarek H., Qatar Genome Program Research Consortium (2024). Analysis of 14,392 Whole Genomes Reveals 3.5% of Qataris Carry Medically Actionable Variants. Eur. J. Hum. Genet. EJHG.

[5] Jeon S., Choi H., Jeon Y., Choi W.-H., Choi H., An K., Ryu H., Bhak J., Lee H., Kwon Y. (2024). Korea4K: Whole Genome Sequences of 4157 Koreans with 107 Phenotypes Derived from Extensive Health Check-Ups. GigaScience.

[6] Miller D.T., Lee K., Abul-Husn N.S., Amendola L.M., Brothers K., Chung W.K., Gollob M.H., Gordon A.S., Harrison S.M., Hershberger R.E. (2023). ACMG SF v3.2 List for Reporting of Secondary Findings in Clinical Exome and Genome Sequencing: A Policy Statement of the American College of Medical Genetics and Genomics (ACMG). Genet. Med. Off. J. Am. Coll. Med. Genet..

[7] Bukaeva A.A., Zaicenoka M., Kiseleva A.V., Drapkina O.D., Meshkov A.N. (2025). Spectrum of Secondary Findings in the DNA Samples from the NMRC TPM Biobank. Med. Genet..

[8] Ramensky V.E., Ershova A.I., Zaicenoka M., Kiseleva A.V., Zharikova A.A., Vyatkin Y.V., Sotnikova E.A., Efimova I.A., Divashuk M.G., Kurilova O.V. (2021). Targeted Sequencing of 242 Clinically Important Genes in the Russian Population From the Ivanovo Region. Front. Genet..

[9] Zhernakova D.V., Brukhin V., Malov S., Oleksyk T.K., Koepfli K.P., Zhuk A., Dobrynin P., Kliver S., Cherkasov N., Tamazian G. (2020). Genome-Wide Sequence Analyses of Ethnic Populations across Russia. Genomics.

[10] Dzhumaniiazova I., Zelenova E., Daniel V., Gusakova M., Kashtanova D., Ivanov M., Blinova O., Yudin V., Matkava L., Mitrofanov S. (2025). Prevalence of Pathogenic and Likely Pathogenic Variants Associated with Cardiovascular Diseases in Russian Adults and Long-Living Individuals. Genes.

[11] Sokolenko A.P., Sokolova T.N., Ni V.I., Preobrazhenskaya E.V., Iyevleva A.G., Aleksakhina S.N., Romanko A.A., Bessonov A.A., Gorodnova T.V., Anisimova E.I. (2020). Frequency and Spectrum of Founder and Non-Founder BRCA1 and BRCA2 Mutations in a Large Series of Russian Breast Cancer and Ovarian Cancer Patients. Breast Cancer Res. Treat..

[12] Imyanitov E.N., Preobrazhenskaya E.V., Shlejkina A.Y., Sokolenko A.P., Anisimova E.I., Ivantsov A.O., Togo A.V. (2018). PALB2 Germ-Line Mutations in Russian Breast Cancer Patients: Identification of Recurrent Alleles and Analysis of Phenotypic Characteristics of the Tumors. Ann. Oncol..

[13] Shulskaya M.V., Shadrina M.I., Bakilina N.A., Zolotova S.V., Slominsky P.A. (2018). The Spectrum of SDHD Mutations in Russian Patients with Head and Neck Paraganglioma. Int. J. Neurosci..

[14] Sokolenko A.P., Venina A.R., Romanko A.A., Belogubova E.V., Sultanbayev A.V., Askarov V.E., Mukhamediarova G.K., Bakaeva E.K., Syomina M.V., Velyukhova T.Y. (2025). 5’UTR Gene Regions in Germline DNA Sequencing Panels: Lessons from the Analysis of Breast and Ovarian Cancer Patients of Tatar and Bashkir Ethnic Origin. Fam. Cancer.

[15] Adler G., Clark J.S., Łoniewska B., Ciechanowicz A. (2011). Prevalence of. Croat. Med. J..

[16] Karunas A.S., Mersiianova I.V., Poliakov A.V., Evgrafov O.V., Khusnutdinova E.K. (2000). Analysis of Mutations and Haplotypes of Polymorphic Markers in Patients with Wilson-Konovalov Disease from Bashkir. Genetica.

[17] Swango K.L., Demirkol M., Hüner G., Pronicka E., Sykut-Cegielska J., Schulze A., Wolf B. (1998). Partial Biotinidase Deficiency Is Usually Due to the D444H Mutation in the Biotinidase Gene. Hum. Genet..

[18] Carmona R., Pérez-Florido J., Roldán G., Loucera C., Aquino V., Toro-Barrios N., Fernández-Rueda J.L., Bostlemann G., López-López D., Ortuño F.M. (2024). Unveiling the Landscape of Reportable Genetic Secondary Findings in the Spanish Population: A Comprehensive Analysis Using the Collaborative Spanish Variant Server Database. medRxiv.

[19] Lenders M., Brand E. (2021). Fabry Disease: The Current Treatment Landscape. Drugs.

[20] Stevens D., Milani-Nejad S., Mozaffar T. (2022). Pompe Disease: A Clinical, Diagnostic, and Therapeutic Overview. Curr. Treat. Options Neurol..

[21] Jolfayi A.G., Kohansal E., Ghasemi S., Naderi N., Hesami M., MozafaryBazargany M., Moghadam M.H., Fazelifar A.F., Maleki M., Kalayinia S. (2024). Exploring TTN Variants as Genetic Insights into Cardiomyopathy Pathogenesis and Potential Emerging Clues to Molecular Mechanisms in Cardiomyopathies. Sci. Rep..

[22] Chan S.H., Bylstra Y., Teo J.X., Kuan J.L., Bertin N., Gonzalez-Porta M., Hebrard M., Tirado-Magallanes R., Tan J.H.J., Jeyakani J. (2022). Analysis of Clinically Relevant Variants from Ancestrally Diverse Asian Genomes. Nat. Commun..

[23] Motwani P., Maurya R.K., Dhwoni, Phadke S.R., Moirangthem A. (2025). Secondary Findings in a Research Cohort: Spectrum and the Indian Perspective. Am. J. Med. Genet. A.

[24] Van Hout C.V., Tachmazidou I., Backman J.D., Hoffman J.D., Liu D., Pandey A.K., Gonzaga-Jauregui C., Khalid S., Ye B., Banerjee N. (2020). Exome Sequencing and Characterization of 49,960 Individuals in the UK Biobank. Nature.

[25] Haer-Wigman L., Van Der Schoot V., Feenstra I., Vulto-van Silfhout A.T., Gilissen C., Brunner H.G., Vissers L.E.L.M., Yntema H.G. (2019). 1 in 38 Individuals at Risk of a Dominant Medically Actionable Disease. Eur. J. Hum. Genet..

[26] Skrahin A., Cheema H.A., Hussain M., Rana N.N., Rehman K.U., Kumar R., Oprea G., Ameziane N., Rolfs A., Skrahina V. (2023). Secondary Findings in a Large Pakistani Cohort Tested with Whole Genome Sequencing. Life Sci. Alliance.

[27] Dewey F.E., Murray M.F., Overton J.D., Habegger L., Leader J.B., Fetterolf S.N., O’Dushlaine C., Van Hout C.V., Staples J., Gonzaga-Jauregui C. (2016). Distribution and Clinical Impact of Functional Variants in 50,726 Whole-Exome Sequences from the DiscovEHR Study. Science.

[28] Kostoulas C., Sesse A., Bouba I., Konitsiotis S., Markoula S., Georgiou I. (2025). Secondary Findings from Exome Sequencing of a Greek Cohort. Curr. Issues Mol. Biol..

[29] Huang Y., Liu B., Shi J., Zhao S., Xu K., Sun L., Chen N., Tian W., Zhang J., Wu N. (2022). Landscape of Secondary Findings in Chinese Population: A Practice of ACMG SF v3.0 List. J. Pers. Med..

[30] Rehm H.L., Berg J.S., Brooks L.D., Bustamante C.D., Evans J.P., Landrum M.J., Ledbetter D.H., Maglott D.R., Martin C.L., Nussbaum R.L. (2015). ClinGen—The Clinical Genome Resource. N. Engl. J. Med..

[31] Anichkov N.M., Kalinina E.Y., Davydova Z.V., Shcherbakova E.V., Yagmurov O.D. (2022). Clinical and Morphological Features of Malignant Hyperthermia: A Rare Case from Practice. Russ. J. Forensic Med..

[32] Majeed S., Johnston C., Saeedi S., Mighton C., Rokoszak V., Abbasi I., Grewal S., Aguda V., Kissoondoyal A., Malkin D. (2024). International Policies Guiding the Selection, Analysis, and Clinical Management of Secondary Findings from Genomic Sequencing: A Systematic Review. Am. J. Hum. Genet..

[33] Nolan J., Buchanan J., Taylor J., Almeida J., Bedenham T., Blair E., Broadgate S., Butler S., Cazeaux A., Craft J. (2024). Secondary (Additional) Findings from the 100,000 Genomes Project: Disease Manifestation, Health Care Outcomes, and Costs of Disclosure. Genet. Med..

[34] Moskovkina E.K. (2024). Legal and Ethical Regulation of Reporting of Secondary Findings. Lex. Genet..

[35] Martin M. (2011). Cutadapt Removes Adapter Sequences from High-Throughput Sequencing Reads. EMBnet.journal.

[36] Li H. (2013). Aligning Sequence Reads, Clone Sequences and Assembly Contigs with BWA-MEM. arXiv.

[37] Poplin R., Chang P.-C., Alexander D., Schwartz S., Colthurst T., Ku A., Newburger D., Dijamco J., Nguyen N., Afshar P.T. (2018). A Universal SNP and Small-Indel Variant Caller Using Deep Neural Networks. Nat. Biotechnol..

[38] Lin M.F., Rodeh O., Penn J., Bai X., Reid J.G., Krasheninina O., Salerno W.J. (2018). GLnexus: Joint Variant Calling for Large Cohort Sequencing. bioRxiv.

[39] Danecek P., Bonfield J.K., Liddle J., Marshall J., Ohan V., Pollard M.O., Whitwham A., Keane T., McCarthy S.A., Davies R.M. (2021). Twelve Years of SAMtools and BCFtools. GigaScience.

[40] McLaren W., Gil L., Hunt S.E., Riat H.S., Ritchie G.R.S., Thormann A., Flicek P., Cunningham F. (2016). The Ensembl Variant Effect Predictor. Genome Biol..

[41] Landrum M.J., Lee J.M., Riley G.R., Jang W., Rubinstein W.S., Church D.M., Maglott D.R. (2014). ClinVar: Public Archive of Relationships among Sequence Variation and Human Phenotype. Nucleic Acids Res..

[42] Chen S., Francioli L.C., Goodrich J.K., Collins R.L., Kanai M., Wang Q., Alföldi J., Watts N.A., Vittal C., Gauthier L.D. (2024). A Genomic Mutational Constraint Map Using Variation in 76,156 Human Genomes. Nature.

[43] Jaganathan K., Kyriazopoulou Panagiotopoulou S., McRae J.F., Darbandi S.F., Knowles D., Li Y.I., Kosmicki J.A., Arbelaez J., Cui W., Schwartz G.B. (2019). Predicting Splicing from Primary Sequence with Deep Learning. Cell.

[44] Tordai H., Torres O., Csepi M., Padányi R., Lukács G.L., Hegedűs T. (2024). Analysis of AlphaMissense Data in Different Protein Groups and Structural Context. Sci. Data.

[45] Manichaikul A., Mychaleckyj J.C., Rich S.S., Daly K., Sale M., Chen W.-M. (2010). Robust Relationship Inference in Genome-Wide Association Studies. Bioinformatics.

[46] Chang C.C., Chow C.C., Tellier L.C., Vattikuti S., Purcell S.M., Lee J.J. (2015). Second-Generation PLINK: Rising to the Challenge of Larger and Richer Datasets. Gigascience.

[47] Auton A., Brooks L.D., Durbin R.M., Garrison E.P., Kang H.M., Korbel J.O., Marchini J.L., McCarthy S., McVean G.A., Genomes Project Consortium (2015). A Global Reference for Human Genetic Variation. Nature.

[48] Bergström A., McCarthy S.A., Hui R., Almarri M.A., Ayub Q., Danecek P., Chen Y., Felkel S., Hallast P., Kamm J. (2020). Insights into Human Genetic Variation and Population History from 929 Diverse Genomes. Science.

[49] Coban-Akdemir Z., White J.J., Song X., Jhangiani S.N., Fatih J.M., Gambin T., Bayram Y., Chinn I.K., Karaca E., Punetha J. (2018). Identifying Genes Whose Mutant Transcripts Cause Dominant Disease Traits by Potential Gain-of-Function Alleles. Am. J. Hum. Genet..

