# Supplementary material for: Carrier Frequencies of Medically Actionable Pathogenic Variants in the Russian Population
Source: Int J Mol Sci. 2026 Jun 13;27(12):5344. doi: 10.3390/ijms27125344 (PMC13299624; doi:10.3390/ijms27125344)
Supplement: Supplementary file 1 [file ijms-27-05344-s001.zip › ijms-4303775-Supplementary Figures.pdf]

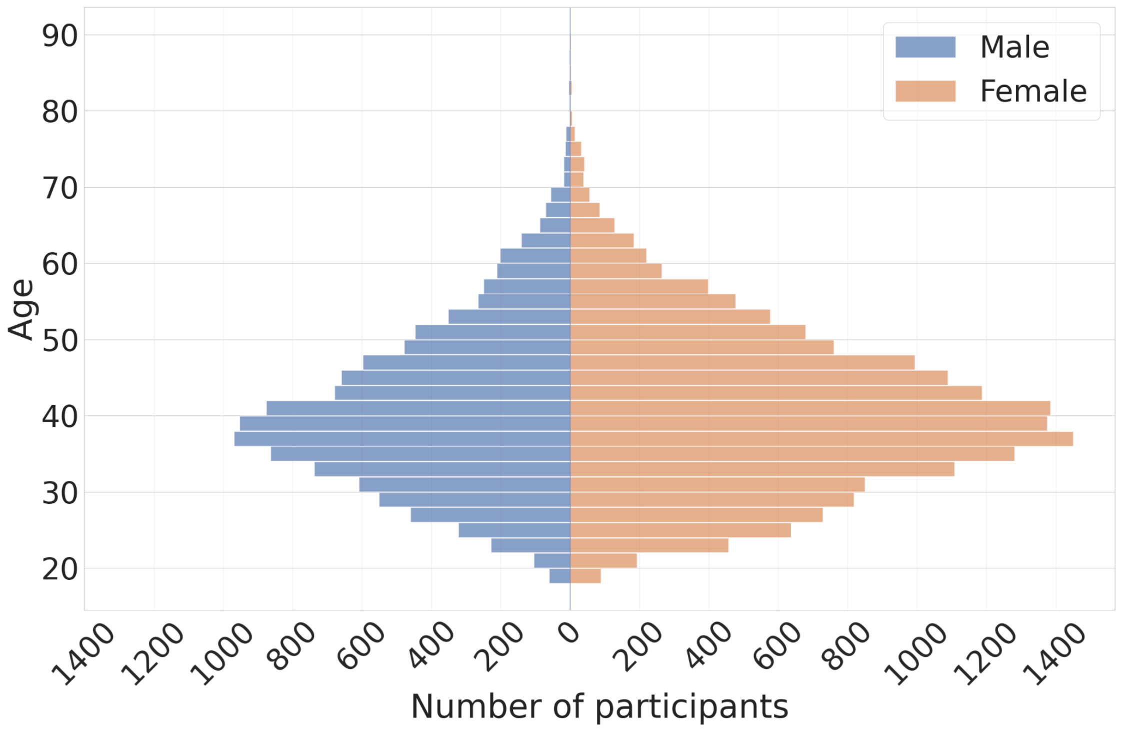

Figure S1. Gender and age data for the NGI cohort.

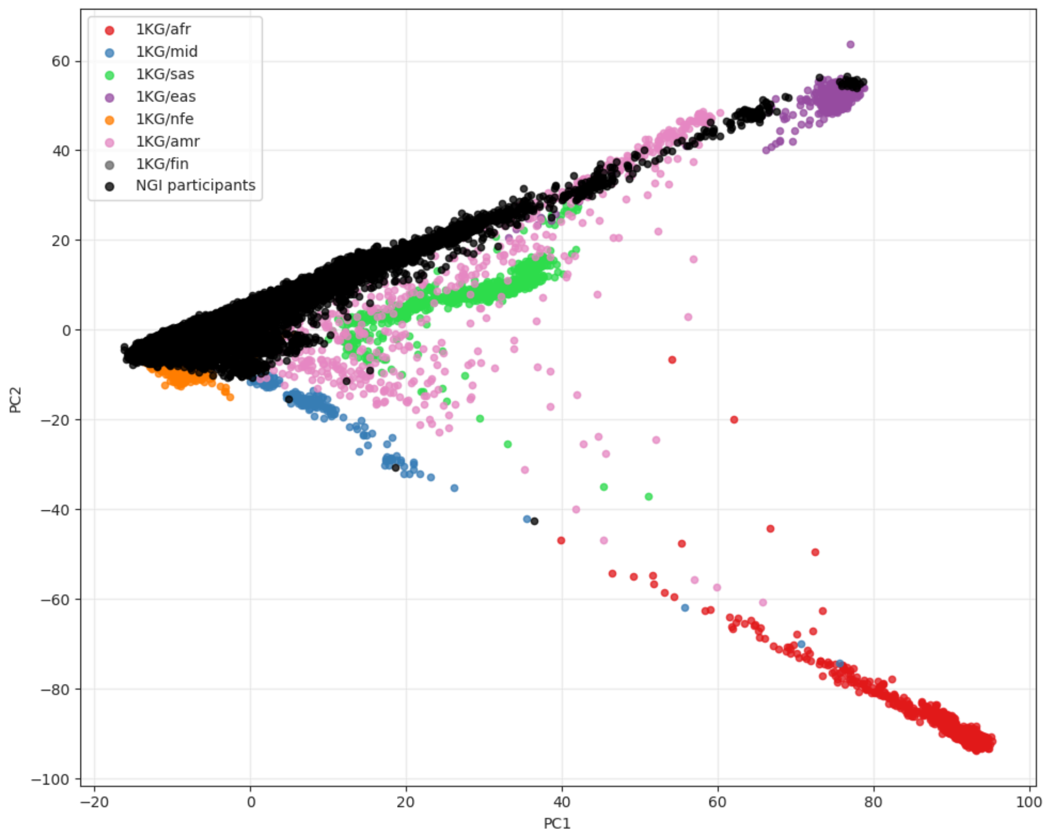

Figure S2. Principal component analysis (PC1 vs PC2) comparing NGI participants with 1000 Genomes Project (1KG) samples (afr: African/African American; amr: Admixed American; eas: East Asian; fin: Finnish; mid: Middle Eastern; nfe: European (non-Finnish); sas: South Asian)**.**

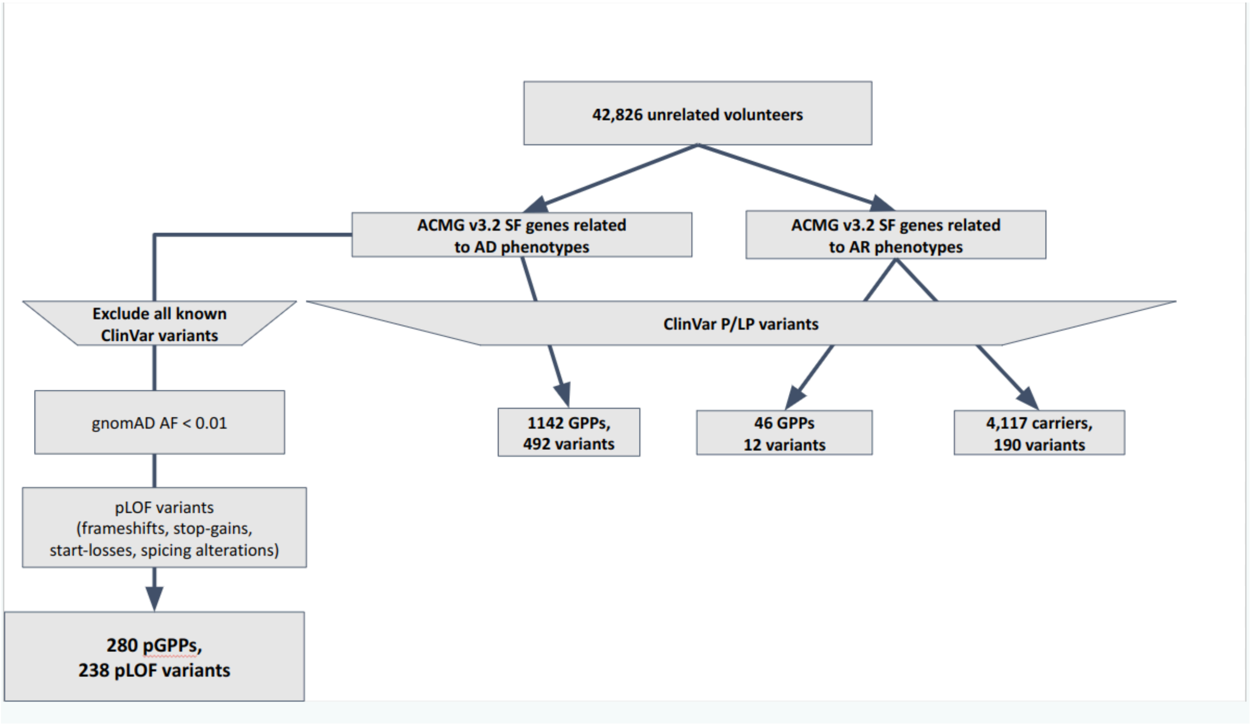

Figure S3. Design of the study. See text for details.

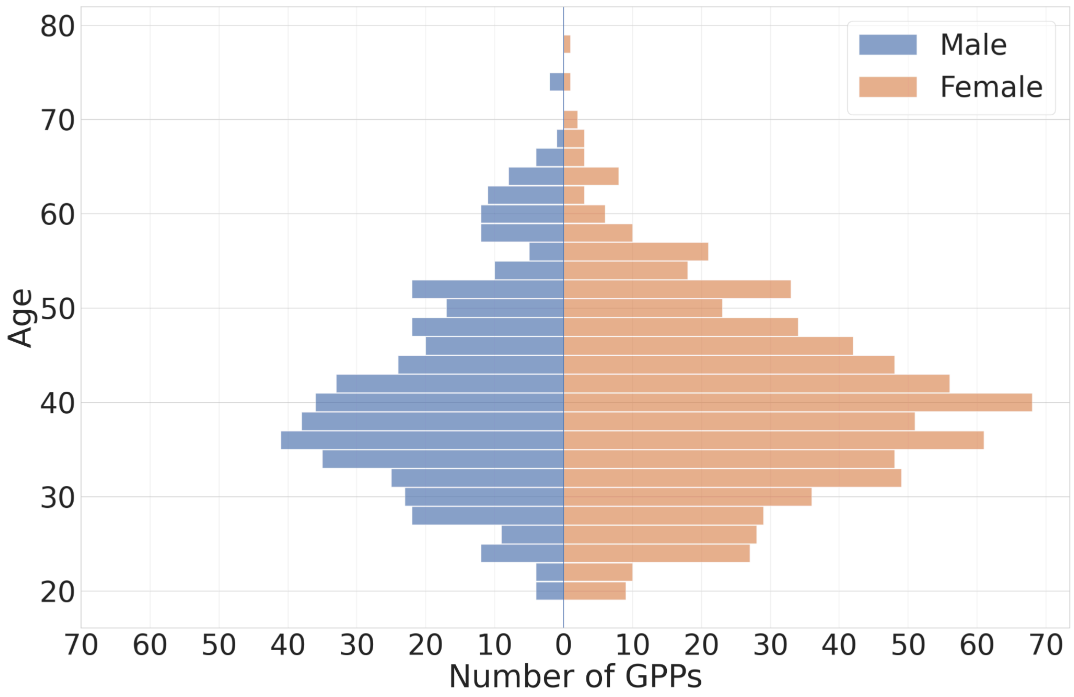

Figure S4. Gender and age data for GPP.

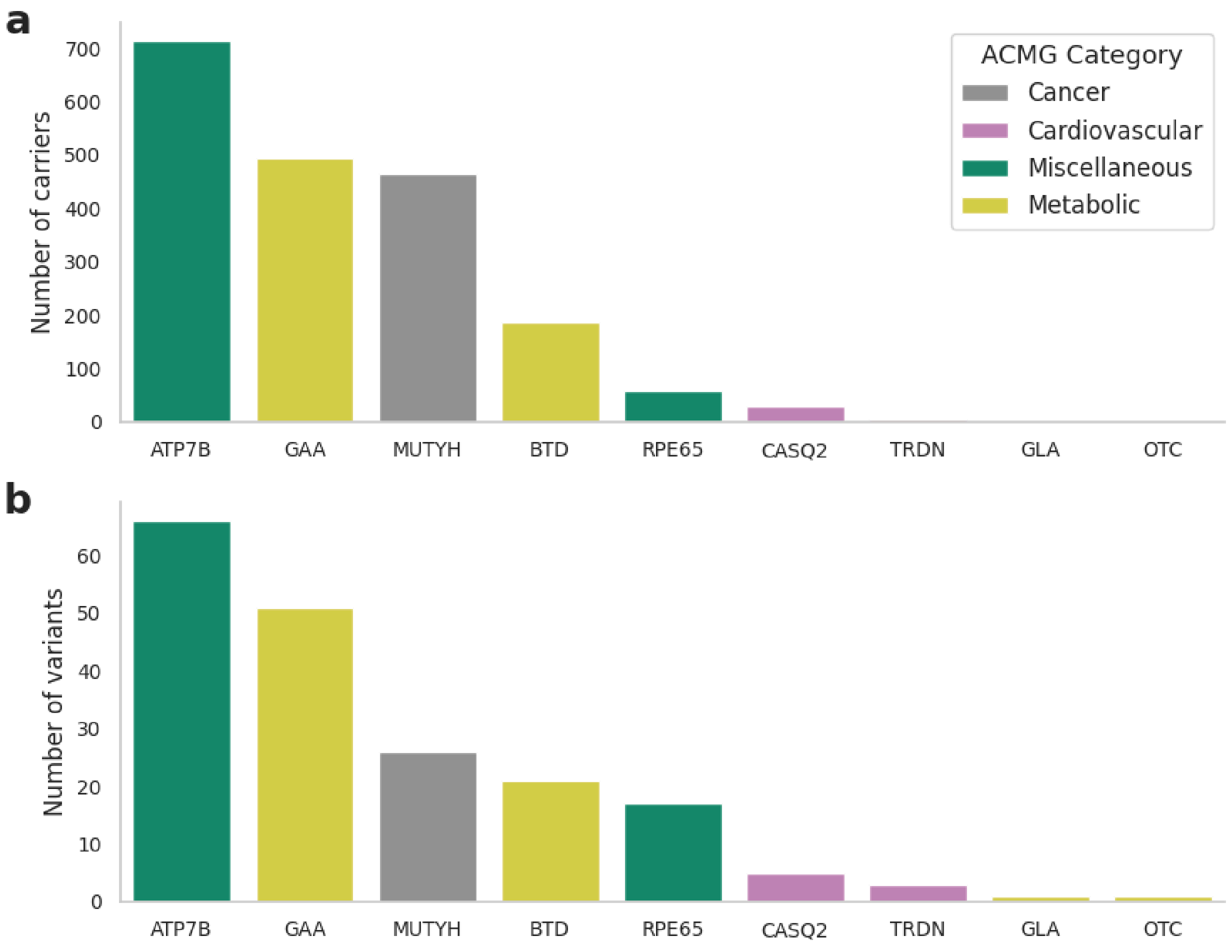

Figure S5. Statistics of carriers of P/LP variants (CPs) in ACMG SF v3.2 genes associated with autosomal recessive phenotypes (a) and the numbers of variants in genes (b) are presented. 2239 CPs with a single reportable variant in the *HFE* gene are not shown.
